# Metal Ion-dependent Heavy Chain Transfer Activity of TSG-6 Mediates Assembly of the Cumulus-Oocyte Matrix*

**DOI:** 10.1074/jbc.M115.669838

**Published:** 2015-10-14

**Authors:** David C. Briggs, Holly L. Birchenough, Tariq Ali, Marilyn S. Rugg, Jon P. Waltho, Elena Ievoli, Thomas A. Jowitt, Jan J. Enghild, Ralf P. Richter, Antonietta Salustri, Caroline M. Milner, Anthony J. Day

**Affiliations:** From the ‡ Wellcome Trust Centre for Cell-Matrix Research and; the ¶ Faculty of Life Sciences, University of Manchester, Oxford Road, Manchester M13 9PT, United Kingdom,; the § Medical Research Council Immunochemistry Unit, Department of Biochemistry, University of Oxford, South Parks Road, Oxford OX1 3QU, United Kingdom,; the ‖ Department of Biomedicine and Prevention, University of Rome Tor Vergata, Rome 00133, Italy,; the ** Department of Molecular Chemistry, University of Aarhus, 8000 Aarhus C, Denmark,; ‡‡ CIC biomaGUNE, 20009 Donostia-San Sebastian, Spain,; the §§ Department of Molecular Chemistry, University Grenoble Alpes and CNRS, 38000 Grenoble, France, and; the ¶¶ Max Planck Institute for Intelligent Systems, 70569 Stuttgart, Germany

**Keywords:** hyaluronan, protein structure, protein-protein interaction, reproduction, site-directed mutagenesis, CUB module structure, TSG-6, cumulus-oocyte complex expansion, heavy chain-hyaluronan complex formation, inter-α-inhibitor

## Abstract

The matrix polysaccharide hyaluronan (HA) has a critical role in the expansion of the cumulus cell-oocyte complex (COC), a process that is necessary for ovulation and fertilization in most mammals. Hyaluronan is organized into a cross-linked network by the cooperative action of three proteins, inter-α-inhibitor (IαI), pentraxin-3, and TNF-stimulated gene-6 (TSG-6), driving the expansion of the COC and providing the cumulus matrix with its required viscoelastic properties. Although it is known that matrix stabilization involves the TSG-6-mediated transfer of IαI heavy chains (HCs) onto hyaluronan (to form covalent HC·HA complexes that are cross-linked by pentraxin-3) and that this occurs via the formation of covalent HC·TSG-6 intermediates, the underlying molecular mechanisms are not well understood. Here, we have determined the tertiary structure of the CUB module from human TSG-6, identifying a calcium ion-binding site and chelating glutamic acid residue that mediate the formation of HC·TSG-6. This occurs via an initial metal ion-dependent, non-covalent, interaction between TSG-6 and HCs that also requires the presence of an HC-associated magnesium ion. In addition, we have found that the well characterized hyaluronan-binding site in the TSG-6 Link module is not used for recognition during transfer of HCs onto HA. Analysis of TSG-6 mutants (with impaired transferase and/or hyaluronan-binding functions) revealed that although the TSG-6-mediated formation of HC·HA complexes is essential for the expansion of mouse COCs *in vitro*, the hyaluronan-binding function of TSG-6 does not play a major role in the stabilization of the murine cumulus matrix.

## Teaser

**Background:** TSG-6 (TNF-stimulated gene-6)-dependent transfer of heavy chains from inter-α-inhibitor onto hyaluronan is critical for ovulation.

**Results:** A calcium ion and chelating glutamate within TSG-6 mediate formation of the covalent heavy chain-TSG-6 intermediate.

**Conclusion:** TSG-6 transferase activity rather than hyaluronan binding drives cumulus expansion.

**Significance:** The role of metal ions in hyaluronan-heavy chain formation has been determined.

## Introduction

In the majority of mammals, ovulation is immediately preceded by the formation of a viscoelastic extracellular matrix by the cumulus cells that surround the oocyte (1, 2). The production of this matrix drives the expansion of the cumulus cell-oocyte complex (COC),^3^ protecting the COC during its expulsion from the follicle, allowing its pickup and transport by the oviduct and providing a large surface area that facilitates sperm capture *in vivo* (3–5). The high molecular weight polysaccharide hyaluronan (HA) is a key structural component of the cumulus matrix; this non-sulfated glycosaminoglycan, composed entirely of repeating disaccharides of glucuronic acid and *N*-acetyl glucosamine, is organized into a cross-linked network during cumulus expansion, providing stability and the required mechanical properties of the COC (6–8). There is compelling evidence that this HA-rich matrix is stabilized through the cooperative action of inter-α-inhibitor (IαI), pentraxin-3, and TSG-6 (TNF-stimulated gene-6) (9–15); all three of these glycoproteins have been implicated as being essential, in the mouse at least (2), because functional depletion/inhibition of any one of them greatly impairs COC expansion, leading to female infertility. Recent biophysical analysis has also demonstrated that human IαI, pentraxin-3, and TSG-6 are sufficient for the formation of a cross-linked matrix in model HA films (16). Most of these components (*i.e.* HA, pentraxin-3, and TSG-6) are produced by the cumulus cells in response to the gonadotropin surge (11, 15, 17, 18), which also leads to the altered permeability of the blood-follicle barrier, allowing IαI to enter from the blood (1).

IαI is composed of three protein chains (bikinun, heavy chain 1 (HC1), and HC2) that are held together covalently by a chondroitin sulfate (CS) chain (19–21); the CS, which contains both sulfated and non-sulfated regions (22–24), is attached to bikunin via a standard glycosaminoglycan linkage, and the HCs are attached to this proteoglycan via ester bonds between their C-terminal aspartic acid residues and C6-hydroxylates of *N*-acetyl galactosamine sugars of the CS chain (25). Importantly, both HC1 and HC2 of IαI can be covalently transferred onto the C6-hydroxyls of the *N*-acetyl glucosamine sugars in HA to form HC·HA, which are sometimes referred to as SHAP-HA (26–29). The formation of these complexes, in which HA is probably decorated with multiple HCs (30), is essential for ovulation/fertilization; deletion of the bikunin gene, which abolishes the biosynthesis of IαI and consequently the production of HC·HA, leads to a lack of cumulus expansion and greatly impaired fertility in mice (9, 10).

Deletion of pentraxin-3 also impairs the incorporation of HA into the COC matrix but does not affect the formation of HC·HA (15). It is likely that pentraxin-3, which has no inherent HA-binding activity (15, 16), contributes to cross-linking of HC·HA complexes via interactions with the attached HC (31); pentraxin-3 is an octameric protein composed of eight identical subunits (connected by disulfide bonds) through which it may bind simultaneously to multiple HCs linking HC·HA complexes together (16, 32–34).

TSG-6 is a 35-kDa (single chain) protein composed mainly of contiguous Link and CUB modules that are flanked by N- and C-terminal sequences of 18 and 29 amino acids, respectively (35, 36). It has been found to be crucial in the formation of HC·HA, and the COCs from *TSG-6*^−/−^ mice (that failed to expand) contained no detectable HC·HA complexes (12). TSG-6 was shown to play a direct role in the transfer of HCs from IαI onto HA via the formation of covalent intermediates (HC1·TSG-6 and HC2·TSG-6) and act as a catalyst in this process (37). These HC·TSG-6 complexes (37, 38) are linked through ester bonds between Ser-28 of TSG-6 (in its N-terminal region) and the C-terminal aspartates of the HCs mentioned above (39); free HCs are unable to form these complexes with TSG-6 (or its individual domains) because this requires the presence of ester bonds connecting HCs to the CS chain of bikunin that are made during the biosynthesis of IαI (see Ref. 37).

Thus, it is clear that the formation of HC·HA involves two sequential transesterification reactions (37, 39, 40). However, beyond this, the molecular bases of HC·TSG-6 complex formation and HC transfer onto HA are not particularly well understood. It is known that these processes are divalent cation-dependent, but there is a lack of consensus on the identity of metal ions required and their locations within the IαI and TSG-6 proteins (37, 40–42); the TSG-6 CUB module has been predicted to contain an Mg^2+^-binding site based on homology with other CUB domains (37). Furthermore, it is far from clear how IαI and TSG-6 interact leading up to the formation of the HC·TSG-6 intermediates (40, 43, 44) or indeed how HA is recognized by these complexes during HC transfer (45). For example, full-length TSG-6 has been shown to interact (non-covalently) with bikunin·CS as well as HC1 and HC2 (40, 44, 46). The former is probably mediated (at least in part) through the binding of the Link module to the CS moiety (40), consistent with its ability to bind to CS and the non-sulfated glycosaminoglycan chondroitin (45, 47); however, the region of TSG-6 that interacts with the HCs is not known. The TSG-6 Link module, for which NMR and x-ray structures are determined (48–50), also mediates the interaction of TSG-6 with HA (see Ref. 51). However, the well characterized HA-binding groove in TSG-6 (45, 49, 52) may not be used for HA recognition by the HC·TSG-6 complexes during HC transfer (45).

TSG-6 also binds directly to pentraxin-3 using a site on the Link module that does not overlap with its HA-binding surface, leading to the hypothesis that pentraxin-3-TSG-6 complexes could cross-link HA chains (15). Although this is apparently not the case for full-length TSG-6 (16), it has not been ruled out that TSG-6 can play a direct structural role in the organization of the cumulus matrix via its HA-binding properties (see Refs. 12, 13, 51, 53, and 54).

It is worthy of mention that HC·HA complexes are also formed in contexts other than ovulation and that, at present, TSG-6 is the only known transferase that can mediate their production. Most likely this is an ancient process in vertebrates, which predates cumulus expansion (55). HC·HA complexes form wherever IαI, TSG-6, and HA come into contact (see Refs. 37 and 55). Given that in most tissues, TSG-6 is only expressed during inflammation (36, 56), it is not surprising that HC·HA complexes are most often associated with inflammatory processes and disease (30, 57–60). Current evidence suggests that decoration of HA with HCs has an important role in modulating cell adhesion and cell phenotype (44, 61, 62) and that certain HC·HA complexes can mediate protective effects (62–66). The precise activity of HC·HA complexes (and whether they are protective or pathological) will probably depend on their exact composition (*e.g.* number and type of HCs, size of HA, etc.) and the identity of other associated structural/signaling molecules (44).

Here we report the tertiary structure of the CUB module from human TSG-6 and the determination of its role in HC transfer. We have also clarified which metal ions are required for HC·TSG-6 complex formation, identifying divalent cation-binding sites in both IαI and TSG-6 that mediate an initial non-covalent interaction. Furthermore, we have demonstrated that it is the HC transferase activity of TSG-6, rather than HA binding, that is crucial for murine COC expansion.

## Experimental Procedures

### 

#### 

##### Production of Recombinant Proteins and HA Oligosaccharides

Full-length human TSG-6 (rhTSG-6) was expressed in *Drosophila* S2 cells and purified as before (67). The Link_TSG6 and CUB_C (Gln-144 allotype) constructs of human TSG-6 (residues 36–133 and 128–277, respectively, of the preprotein (35)) were expressed in *Escherichia coli*, refolded, and purified as described previously (68–70). Recombinant human heavy chain 1 (rHC1) was produced as detailed previously (44). Mutations in CUB_C and rHC1 were introduced into the expression vectors by QuikChange mutagenesis (Agilent Technologies, Cheadle, UK), essentially following the manufacturer's recommendations. Mutations in rhTSG-6 were introduced into the expression vector using the Transformer site-directed mutagenesis kit (Clontech, Palo Alto, CA), following the manufacturer's guidelines (see Refs. 67 and 71). HA 14-mers (HA_14_) and biotinylated HA decasaccharide (bHA_10_) were made as described previously (in Refs. 72 and 73, respectively).

##### Crystallization of CUB_C and Determination of CUB Module Structure

Initial crystallization conditions were obtained using the SM1 screen from Qiagen (Manchester, UK), using the sitting drop vapor diffusion technique at room temperature. A focused screen around these initial conditions yielded 30-μm crystals with a tetragonal bipyramidal morphology. Seeds derived from these crystals were streaked into drops containing 1 μl of 5 mg/ml CUB_C mixed with 1 μl of mother liquor composed of 100 mm HEPES, pH 7.5, 22% (v/v) PEG 1000, and 200 mm MgSO_4_, yielding larger crystals.

Data to 2.3 Å were collected from 250 × 250 × 250-μm crystals, cryoprotected using Paratone-N oil (Molecular Dimensions, Cambridge, UK), on a Rigaku-007 rotating anode x-ray generator and an R-axis IV image plate detector. The data were indexed and integrated using the Mosflm program (74). Point group analysis using the POINTLESS program (75) indicated either P4_3_2_1_2 or P4_1_2_1_2 symmetry. Data were scaled and reduced using the Scala and Truncate programs (76). Matthews coefficient analysis indicated the presence of one monomer in the asymmetric unit. Initial phases were obtained by molecular replacement using the Molrep program (77), and a homology model of CUB, based upon MAp19 coordinates (Protein Data Bank code 1SZB) (78), was generated using the PHYRE server (79). The molecular replacement solution confirmed P4_3_2_1_2 symmetry. This model was then refined to convergence with alternative rounds of restrained maximum likelihood refinement using REFMAC (80) and manual rebuilding using the Coot program (81). Final processing statistics are shown in Table 1. Only the CUB module (residues 128–249) was visible in the crystal structure with no electron density seen for the last 28 amino acid residues, although this region was verified as present in the crystals by SDS-PAGE (not shown). The last residue observed in the electron density is Pro-249, which protrudes into a large solvent channel; this might allow the C-terminal region to adopt a range of conformations, thus rendering it essentially invisible to crystallography.

##### HC·TSG-6 and HC·HA Complex Formation

The effects of rhTSG-6 mutagenesis on the formation of HC·TSG-6 and HC·HA complexes were determined using the assays described previously (37, 45). Briefly, in the standard experiment, 80 μg/ml (2.7 μm) rhTSG-6 and 320 μg/ml (1.8 μm) IαI, purified from human serum (19, 82), were incubated together in 20 mm HEPES, pH 7.5, 150 mm NaCl, 5 mm MgCl_2_ in a total volume of 25 μl for 2 h at 4 °C; this was done either in the absence (“complex formation”) or presence (“HC transfer”) of 40 μg/ml HA_14_ or bHA_10_. In complex formation experiments where EGTA/Ca^2+^ was also added, only 1 mm MgCl_2_ was used, which is sufficient for full activity (37); when the pH was varied, sodium acetate (pH 4.0 or 5.0) or MES (pH 6.0 or 6.5) were used instead of HEPES (pH 7.0, 7.5, or 8.0). Samples (7.5 μl of reaction mixture) were then run on 10% (v/v) Tris-Tricine/SDS-polyacrylamide gels after reduction with 5% (v/v) β-mercaptoethanol in SDS protein sample buffer (5 min at 100 °C) and stained with Coomassie Blue. Alternatively, HC·TSG-6 complexes were visualized by Western blotting using a rabbit anti-human polyclonal antibody (RAH-1) raised against TSG-6 (see Ref. 37); HC·bHA_10_ complexes (16) were detected with streptavidin-conjugated Alexa 488 (Invitrogen) on a LI-COR Odyssey system, and band intensities were quantified by ImageJ software. All gels and blots shown are representative of at least three independent experiments.

##### Surface Plasmon Resonance

Surface plasmon resonance (SPR) experiments were carried out using a BiaCore 3000 or T-200 (GE Healthcare). CUB_C proteins (WT or E183S) and WT rhTSG-6 were coupled to a BiaCore CM5 chip (to give ∼1000 response units) via standard amine-coupling chemistry (EDC-NHS); here CUB_C (50 μg/ml) and WT rhTSG-6 (10 μg/ml) were immobilized in 10 mm sodium acetate, pH 4.0, and 10 mm MES, pH 6.0, respectively, at a flow rate of 10 μl/min. Then rHC1 (WT or D298A) was injected over the chip surface (30 μl/min) at a range of concentrations in 20 mm HEPES, 150 mm NaCl, 0.05% (v/v) Tween 20, pH 7.4 (HBS-T), with/without EDTA (see Table 2); as described above, in the absence of EDTA (“standard” buffer conditions) the “as purified” CUB_C proteins contain Ca^2+^ ions. In other experiments, rHC1 (WT or D298A) was immobilized on a C1 chip (as described in Ref. 44; *i.e.* at 10 μg/ml in 10 mm sodium acetate, pH 5.5, at 10 μl/min), and Link_TSG6 was flowed over at a range of concentrations in HBS-T. All SPR experiments were performed in duplicate or triplicate, and numerical values (mean ± S.E. in Table 2) were determined from multicycle kinetics, where data were fitted to a 1:1 Langmuir model using the BiaEval T-200 software; fitting of data to a bivalent analyte model did not improve any of the fits.

##### Intrinsic Fluorescence

CUB_C protein was incubated in 20 mm HEPES-HCl, 150 mm NaCl, pH 7.4 (HBS) with 10 mm EDTA and 10 mm EGTA before buffer exchange into HBS with 2 μm EDTA and 2 μm EGTA (to remove metal ions and prevent metal ion scavenging, respectively). Intrinsic fluorescence spectra were recorded on 3 μm CUB_C in the absence/presence of 20 μm metal ions on a Jasco (Dunmow, UK) FP750 spectrofluorometer compared with “as purified” CUB_C in HBS alone; the CaCl_2_, MgCl_2_, and MnCl_2_ used (Ultrapure, Sigma-Aldrich) were ultrahigh purity (99.999% (w/w) trace metal basis). The excitation wavelength was set at 280 nm, and emission spectra were recorded between 300 and 400 nm with excitation/emission wavelength slit widths of 4 nm. All measurements were performed in triplicate and averaged after buffer subtraction.

Additionally, the fluorescent intensity of the tryptophan peak at 330 nm was determined (as above) for 2 μm CUB_C in HBS (containing 2 μm EGTA as a metal ion scavenger) at a range of CaCl_2_ concentrations (0–40 μm). Data were fitted by non-linear regression to a one-site model; the Ca^2+^ ion concentrations used in the fitting were not corrected to take account of the added EGTA.

##### Nuclear Magnetic Resonance Spectroscopy

One-dimensional ^1^H NMR spectra were collected on 0.1 mm CUB_C (WT or E183S) in PBS (pH 6.5) using a Bruker (Coventry, UK) Avance 600-MHz spectrometer (equipped with a ^1^H-^13^C/^15^N TXI cryoprobe with *z*-gradients) at 25 °C in the absence (“as purified”) or presence of EGTA (5 mm or as indicated) and/or CaCl_2_ (10 mm).

##### HA-binding Assay

The HA-binding activity of rhTSG-6 (WT and mutants) was determined using a microtiter-based assay employing biotinylated HA as described previously (83). Briefly, TSG-6 proteins were immobilized at various concentrations on Maxisorb plates (Nunc, Fisher, Loughborough, UK) and incubated with biotinylated HA (12.5 ng/well) in 50 mm sodium acetate, 100 mm NaCl, 0.05% (v/v) Tween 20, pH 6.0; bound biotinylated HA was detected with ExtraAvidin alkaline phosphatase, and plates were developed using disodium *p*-nitrophenyl phosphate. All absorbance measurements (*A*_405 nm_) were corrected by subtracting values from uncoated control wells. Mean data ± S.E. were derived from two independent experiments performed in quadruplicate (*n* = 8).

##### COC Expansion Assays

Experiments involving animals were approved by the institutional animal care and use committee and carried out according to the Italian and European rules (D.L. 116/92; EEC Council Directive 86/609; European Directive 2010/63/EU). COC isolation, culture and *in vitro* expansion were carried out essentially as described before (12, 84). Briefly, adult BALB/c female mice deficient for TSG-6 (12) or wild type (*TSG-6*^+/+^) controls were injected with 5 IU of pregnant mares serum gonadotropin (Folligon, MSD Italia, Rome, Italy) and sacrificed 44–48 h later. COCs were isolated from large antral follicles in Eagle's minimum essential medium (Gibco Invitrogen, Milan, Italy) containing 25 mm HEPES, 0.1% (w/v) BSA (Sigma-Aldrich), and 50 ng/ml gentamycin (Gibco Invitrogen). Compact COCs were cultured, under mineral oil (Sigma-Aldrich), in 20-μl droplets of Eagle's minimum essential medium supplemented with 5% (w/v) fetal bovine serum (Gibco Invitrogen), 3 mm glutamine (Sigma-Aldrich), 0.3 mm sodium pyruvate (Sigma-Aldrich), and 50 ng/ml gentamycin in the presence of 3 ng/ml EGF (Life Technologies), at 37 °C with 5% (v/v) CO_2_ in humidified air for 16 h. TSG-6 proteins were added to the medium at the beginning of culture, at the concentrations indicated in “Results.”

## Results

### 

#### 

##### Structure of TSG-6 CUB Module Reveals a Ca^2+^ Ion-binding Site

**FIGURE 1.**
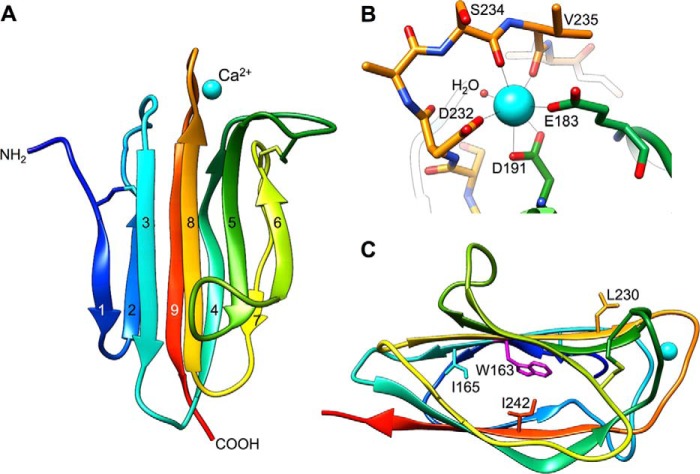
**Structure of TSG-6 CUB module.** *A*, cartoon representation of the crystal structure of the CUB module from human TSG-6, *colored* from *blue* (N terminus) to *red* (C-terminus) with the calcium ion in *cyan. B*, detailed view of the calcium ion-binding site, with ligating residues highlighted. *C*, side view of TSG-6 CUB module, *colored* as in *A*, showing the positions of side chains for Trp-163, Ile-165, Leu-230, and Ile-242. Figures were created using UCSF Chimera.

To test our prediction that a functionally important metal ion-binding site is present within human TSG-6 (37, 85), we determined the crystal structure of its CUB module to 2.3 Å (Fig. 1*A* and Table 1); this was based on the crystallization of the human CUB_C domain (*i.e.* the CUB module and the C-terminal 29 amino acid residues), which we had previously shown by NMR spectroscopy to be folded (70). This revealed it to have a classical CUB-type jelly roll fold, with a greater degree of similarity to the metal ion-binding subclass of CUB domains (C1s, MASPs) compared with the non-metal ion-binding spermadhesins (86), and, consistent with this, it contained a divalent cation at the site we had predicted (Fig. 1).

**TABLE 1:** **Crystallographic data and refinement statistics**

Parameters	Values
Source	CuKα
Space group	P4_3_2_1_2
Unit cell parameters	*a* = *b* = 56.93 Å, *c* = 112.57 Å, α = β = γ = 90°
Resolution range (Å)	40–2.3 (2.42–2.35)*^a^*
No. of unique reflections	8616
Redundancy	5.4 (2.5)
*I*/σ*I*	8.5 (1.3)
Completeness (%)	98.7 (92.1)
*R*(p.i.m.) (%)	4.9 (51.8)
*R*_Cryst_/*R*_free_ (%)	19.1/22.8
Root mean square deviation, bonds (Å)/angles (degrees)	0.007/1.113
Average ADP*^b^* protein/solvent	13.6/39.6

**Ramachandran plot (%)**	
Most favored	94.9
Additionally allowed	3.4
Outliers	1.7

*^a^* Numbers in parentheses represent the values for the highest resolution shell.

*^b^* Atomic displacement parameter.

The metal ion-binding site in the TSG-6 CUB module consists of seven oxygen ligands with pentagonal bipyramidal coordination geometry. This is composed of the main chain carbonyl groups of Ser-234 and Val-235, monodenate coordination with side chain carboxylate groups of Glu-183 and Asp-232, and bidentate coordination with the carboxylate of Asp-191 and a buried water molecule (Fig. 1*B*), an arrangement highly suggestive of Ca^2+^ (or perhaps Mn^2+^). This binding site is very similar to that found in other CUB modules (78, 87), which can be capable of accommodating both Mg^2+^ and Ca^2+^ ions. Based on our previous finding that the formation of HC·TSG-6 complexes was dependent on the presence of magnesium ions, we had expected to find a Mg^2+^ rather than Ca^2+^ at this site (37). However, no magnesium ion-binding sites were discovered during the refinement and analysis of the TSG-6 CUB module structure, despite the presence of 200 mm MgSO_4_ in the crystallization conditions. Analysis of the structure using the “WASP” server (88) confirmed the absence of Mg^2+^-binding sites in the CUB module structure and confirmed that none of the modeled water molecules were magnesium ions.

**FIGURE 2.**
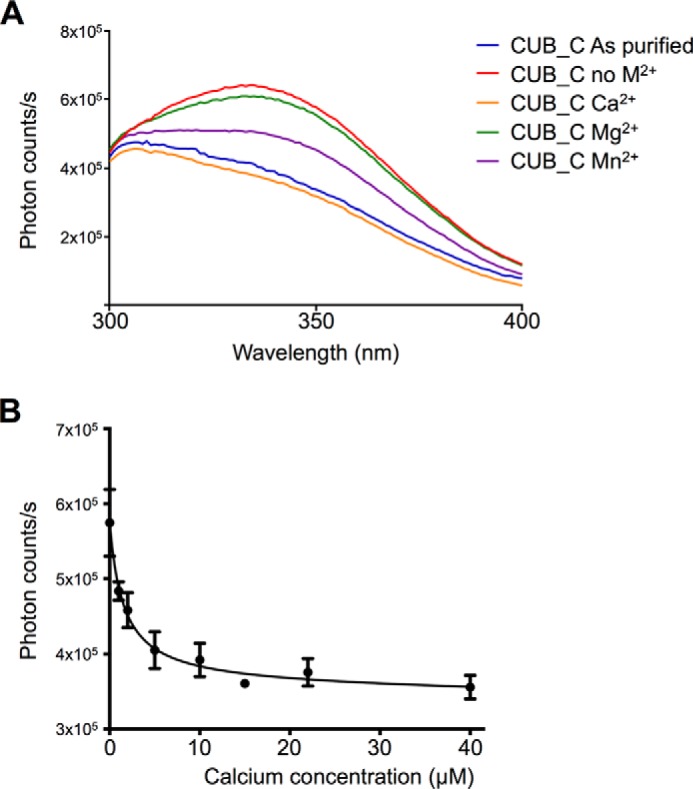
**The TSG-6 CUB_C domain binds to calcium and manganese ions.** *A*, intrinsic fluorescence spectra of CUB_C in HBS (*As purified*) or HBS with 2 μm EDTA, 2 μm EGTA in the absence (*no M^2^*^+^) and presence of 20 μm divalent cations; spectra are averaged from three independent experiments after buffer subtraction. *B*, tryptophan fluorescence intensity values for 2 μm CUB_C (in HBS, 2 μm EGTA) were determined (in triplicate) in the presence of 0–40 μm CaCl_2_ without subtraction of background fluorescence; these data (mean ± S.D.) were plotted against Ca^2+^ ion concentration and analyzed by non-linear regression to a one-site binding model (using the formula, fluorescence intensity = *R*_min_·[Ca^2+^]/*K_D_*·[Ca^2+^] in GraphPad Prism), yielding a *K_D_* value of 1.6 ± 0.6 μm. No errors are shown for the data point at 15 μm Ca^2+^ because these are within the range covered by the *data symbol*.

Intrinsic fluorescence spectroscopy showed that the addition of 20 μm CaCl_2_ to metal ion-free CUB_C (3 μm) in physiological salt/pH conditions results in a significant quenching of tryptophan fluorescence at 330 nm, leading to tyrosine fluorescence becoming visible at ∼305 nm (Fig. 2*A*); because there is only one tryptophan in CUB_C, it can be concluded that Trp-163 (Fig. 1*C*) is sensitive to Ca^2+^ ion binding through an altered conformation and/or altered solvent accessibility of the CUB module. Whereas the addition of MgCl_2_ has essentially no effect on the intrinsic fluorescence spectrum, it can be seen that Mn^2+^ binds to CUB_C, but the observed change in fluorescence is less pronounced than with CaCl_2_, indicative of weaker (or differential) binding (Fig. 2*A*). Non-linear regression analysis of the tryptophan intensities for 2 μm CUB_C at a range of added CaCl_2_ concentrations (0–40 μm) determined a binding affinity of 1.6 ± 0.6 μm for the interaction of CUB_C with Ca^2+^ ions (Fig. 2*B*). In Fig. 2*A*, the finding that the spectra in the presence of Ca^2+^ were essentially identical to those collected on the “as purified” CUB_C domain (with no added metal ions or chelators) indicates that following purification, the protein already contains bound calcium.

**FIGURE 3.**
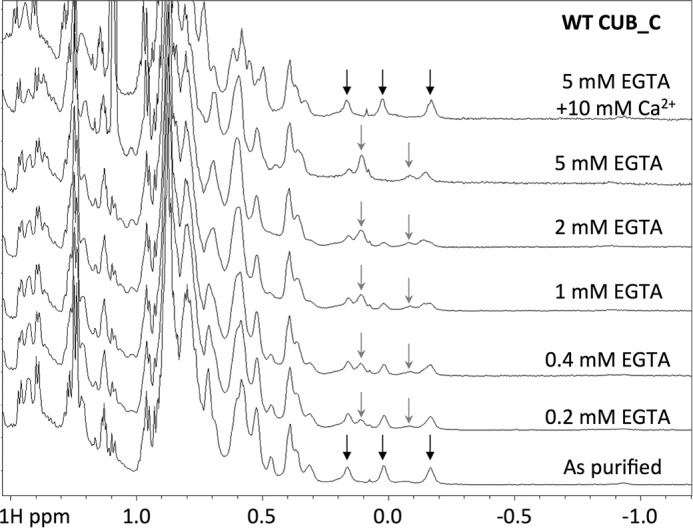
**NMR spectra of WT CUB_C in the absence/presence of calcium.** One-dimensional ^1^H NMR spectra were collected for WT CUB_C in the absence (*As purified*) or presence of added Ca^2+^ ions and/or EGTA. The *black arrows* denote the positions of high field-shifted methyl protons that are consistent with a WT fold, where these are perturbed (*gray arrows*) upon removal of calcium.

To further explore calcium ion binding, we carried out one-dimensional ^1^H NMR measurements on CUB_C in different concentrations of EGTA and calcium (Fig. 3). Upon the addition of EGTA to “as purified” CUB_C, there were no major changes in the NMR spectrum, with only subtle alterations observed in the position/intensity of high field-shifted methyl peaks, which were reversed upon the addition of excess Ca^2+^ ions. Calculations of ^1^H chemical shifts from the CUB module crystal structure using ShiftX (89) predict that the most high field-shifted resonances correspond to the methyl protons of Ile-165, Leu-230, and Ile-242; of these, Leu-230 is in close proximity to aromatic residues in the vicinity of the Ca^2+^ ion-binding site, and Ile-165 and Ile-242 are close to Trp-163 that is affected by Ca^2+^ binding (see Fig. 1*C*). Overall, these data provide evidence that Ca^2+^ is not required for stabilization of the TSG-6 CUB module fold but rather plays a role in the local organization of loops surrounding the calcium ion-binding site (see Fig. 1, *A* and *B*) and perhaps also has some effect on the stability of the protein core. The similarity of the NMR spectrum for the “as purified” protein to that for CUB_C in the presence of excess Ca^2+^ ions (Fig. 3) demonstrates that following purification, the CUB_C protein is fully calcium-bound.

##### Glu-183 in the CUB Ca^2+^ Ion-binding Site Is Involved in HC·TSG-6 Complex Formation

**FIGURE 4.**
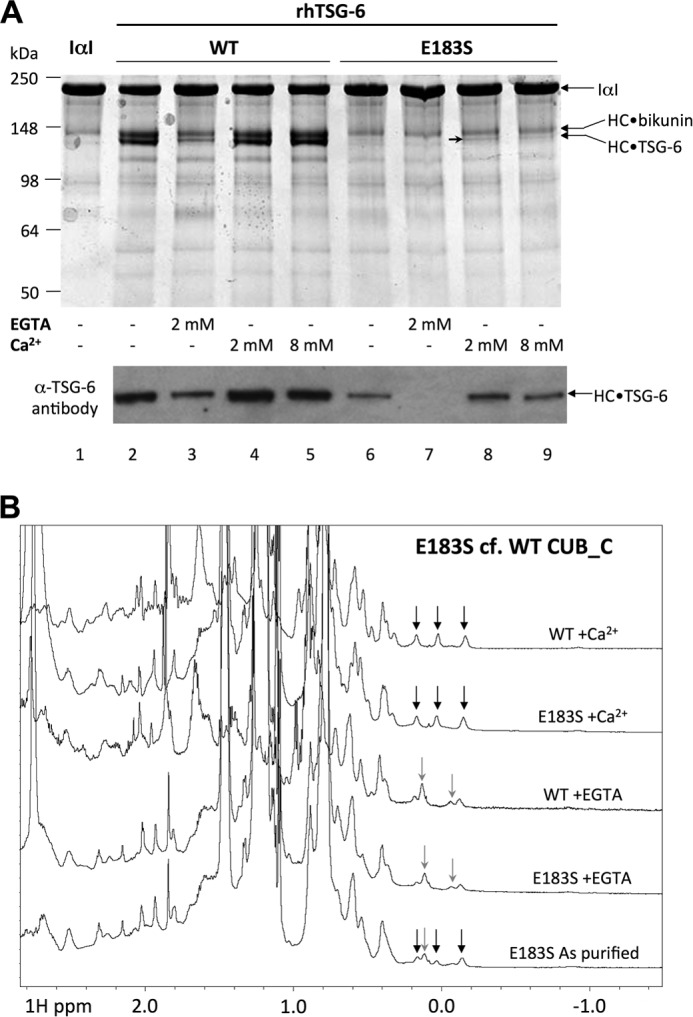
**Structural and functional analyses of WT and E183S TSG-6 in the absence/presence of calcium.** *A*, SDS-PAGE (*top*) and Western blot (*bottom*) analyses reveal that E183S mutation severely diminishes HC·TSG-6 complex formation activity of rhTSG-6 and the production of HC·bikunin, which is a by-product of the reaction (see Ref. 37); the *arrowhead* indicates the faint HC·TSG-6 band generated with the E183S mutant of TSG-6 in the presence of added Ca^2+^. The gel/blot shown is representative of three independent experiments. *B*, one-dimensional ^1^H NMR spectra of WT and E183S CUB_C in the absence or presence of added Ca^2+^ ions (10 mm) or EGTA (5 mm). The *black* and *gray arrows* show high field-shifted methyl protons as described in the legend to Fig. 3; the spectrum for the “as purified” E183S contains a mixture of these resonances, indicating that in this mutant, the Ca^2+^-binding site is only partially occupied with metal ions.

Two residues of TSG-6 involved in chelating calcium (Glu-183 and Asp-232; Fig. 1*B*) were mutated in order to determine the role of the Ca^2+^ ion-binding site in the formation of HC·TSG-6 complexes. E183S and D232A mutants, made in the context of the recombinant full-length protein (rhTSG-6), were found to have greatly diminished ability to form HC·TSG-6 compared with the wild-type (WT) protein based on visualization of this complex on SDS-PAGE (Fig. 4*A*; data not shown for Asp-232); Western blot analysis revealed a low level of activity for E183S (see *lanes 2* and *6* in Fig. 4*A* for WT and E183S, respectively, under standard assay conditions).

Analysis of equivalent mutations in the CUB_C domain by intrinsic fluorescence spectroscopy revealed that alteration of Asp-232 to alanine compromises the folding of the protein (explaining its lack of activity), whereas the E183S mutant remains folded (data not shown). Analysis by NMR confirmed that E183S is folded (Fig. 4*B*). It also demonstrated that although the E183S and WT proteins have very similar spectra in the presence of EGTA, E183S in the absence of any added EGTA or added Ca^2+^ (*i.e.* “as purified”) is a mixture of calcium-bound and calcium-free material. This is indicative of reduced Ca^2+^ ion-binding affinity for E183S compared with WT CUB_C, which is not surprising given the direct role of Glu-183 in calcium chelation (Fig. 1*B*). Consistent with this, EGTA treatment effectively abolished HC·TSG-6 complex formation in E183S, whereas WT rhTSG-6 retained some activity (Fig. 4*A*). Interestingly, in the presence of excess Ca^2+^ (10 mm), the high field region of the NMR spectrum for E183S closely resembles that of the WT CUB_C domain (Fig. 4*B*), revealing that under these conditions, it can become fully calcium-bound and attain a fold essentially identical to WT. However, in functional assays, the addition of calcium ions only increased the amount of HC·TSG-6 product formed for E183S to ∼5% of that seen for the WT protein (estimated from SDS-PAGE band intensity; Fig. 4*A*, *lanes 8* and *9*). These findings strongly suggest that the side chain of Glu-183 plays an important role in the formation of the covalent complex between TSG-6 and HC that is only partly dependent on its chelation of Ca^2+^.

##### Both CUB_C and Link Domains of TSG-6 Interact with IαI HCs

**FIGURE 5.**
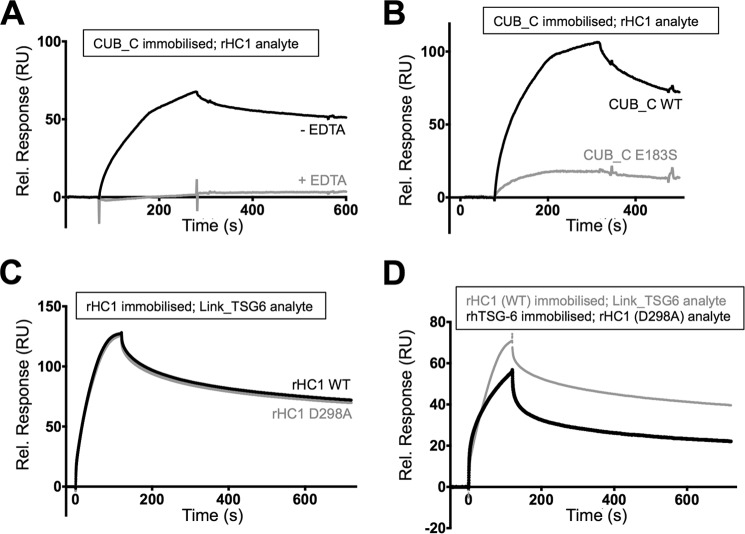
**Metal ion-dependent interaction of TSG-6 with IαI heavy chains is mediated by its CUB_C domain.** *A–D*, comparison of SPR sensorgrams for the interactions of HCs with TSG-6 (WT and mutant proteins in the absence/presence of metal ions); full SPR data sets are provided in Fig. 6 with derived mean numerical values shown in Table 2. *A*, SPR sensorgrams for interactions of immobilized CUB_C (as purified; *i.e.* containing Ca^2+^) with WT rHC1 (20 nm) in the absence or presence of 0.5 mm EDTA. *B*, SPR sensorgrams for interactions of immobilized CUB_C (WT or E183S) with 50 nm rHC1. *C*, SPR sensorgrams for interactions of immobilized rHC1 (WT or D298A) with 200 nm Link_TSG6; the concentration of rHC1 was 50 nm compared with 20 nm in *A. D*, SPR sensorgrams for interactions of immobilized rHC1 (WT) with Link_TSG6 and immobilized rhTSG-6 with rHC1 (D298A); the concentration of Link_TSG6 is 100 nm compared with 200 nm in *C. RU*, response units.

**FIGURE 6.**
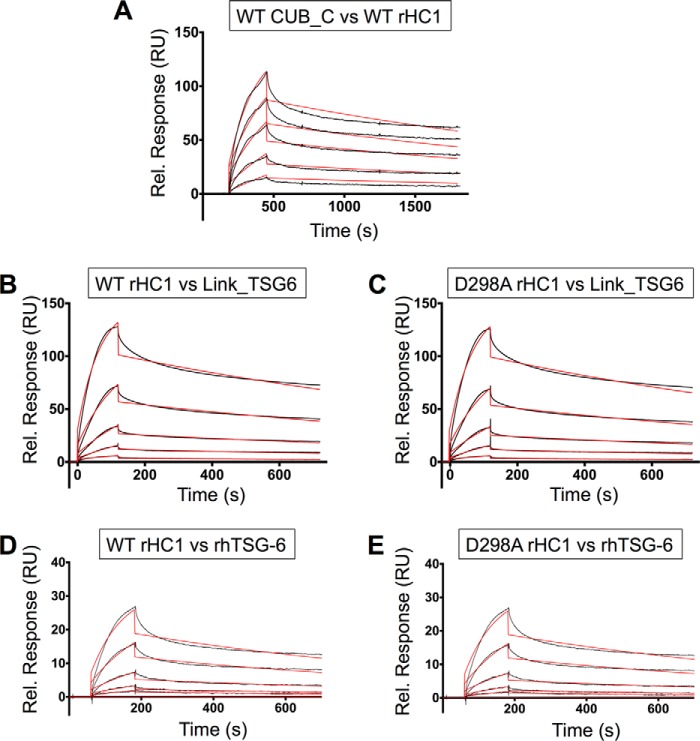
**SPR analyses of the interactions of rHC1 with TSG-6.** SPR sensorgrams (*black lines*) for the interactions of immobilized WT CUB_C with WT rHC1 (*A*), immobilized WT (*B*) and D298A (*C*) rHC1 with Link_TSG6, and immobilized WT (*D*) and D298A (*E*) rHC1 with rhTSG-6; concentrations were 5, 10, 20, 25, and 30 nm for WT rHC1 in *A* and 18.75, 37.5, 75, 150, and 300 nm for analytes in (*B–D*). Fits from the Langmuir 1:1 model are shown in *red*. Data are representative of three independent experiments (see Table 2 for derived numerical values). Despite some apparent biphasic behavior in the interactions, there were no marked improvements in the fits when a bivalent analyte model was applied; moreover, fits with the Langmuir 1:1 model yielded acceptable χ^2^ values (Table 2), indicating that the derived values represent reasonable estimates of the affinity and kinetics for these interactions. *RU*, response units.

Given the observation that the activity of E183S can be partially restored with excess Ca^2+^ (Fig. 4*A*), we reasoned that it is unlikely that this residue has a direct catalytic role in HC·TSG-6 formation but rather that it might participate in substrate (*i.e.* heavy chain) recognition. In order to test this, we looked at binding of CUB_C to rHC1 using SPR. As shown in Fig. 5*A*, WT CUB_C interacted with rHC1 in the presence of Ca^2+^ (*K_D_* = 2.1 nm; see Table 2) but did not bind in EDTA; in Fig. 5, pairs of sensorgrams are illustrated at a single common analyte concentration (*i.e.* for the purposes of easy comparison), where the full SPR data sets are provided in Fig. 6, *A–E*. Importantly, the E183S mutant had greatly impaired binding to rHC1 (Fig. 5*B*), providing evidence that this amino acid residue does make a contribution to the non-covalent interaction between TSG-6 and HCs, which is believed to precede formation of the covalent HC·TSG-6 complex (40, 44). Furthermore, we found that a D298A mutation in the context of rHC1, which forms part of a conserved MIDAS site (37) and abolishes Mg^2+^ ion binding within the HC1 von Willebrand factor A domain (*i.e.* based on x-ray crystallography of WT and D298A mutant of rHC1),^4^ is unable to bind CUB_C (Table 2). This strongly suggests that metal ion-binding sites in both the CUB module and von Willebrand factor A domains contribute to the interaction between TSG-6 and HCs.

**TABLE 2:**
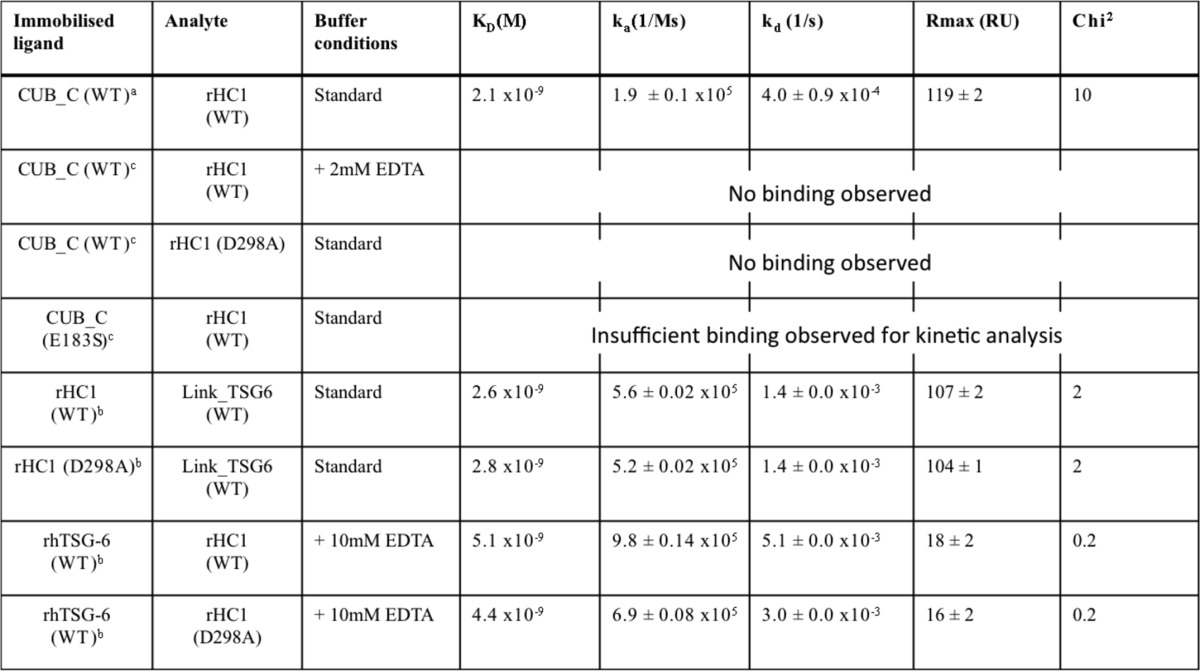
**Surface plasmon resonance analyses and parameters**

*^a,b^* All values (mean ± S.E.) are based on multicycle kinetics from experiments performed in duplicate (*a*) or triplicate (*b*) (analyte at 0–30 nm and 0–300 nm, respectively).

*^c^* Experiments performed at a single concentration (20 nm) in triplicate.

Full-length TSG-6 has been shown to be able to bind non-covalently to HC1 and HC2 in a metal ion-independent manner (44), which is inconsistent with the above analysis. Therefore, we reasoned that another region of TSG-6 might also contribute to the binding to HCs. SPR with the Link module of TSG-6 (Link_TSG6, which does not have any metal ion binding sites (50)) showed that this domain can interact with rHC1 (both WT and D298A; Figs. 5*C* and 6 (*B* and *C*)), where this had very similar binding kinetics to the interaction of rhTSG-6 with the D298A mutant (Figs. 5*D* and 6*E* and Table 2). Thus, we conclude that full-length TSG-6 can interact with HC1 at two distinct sites: a metal ion-independent interaction with the Link domain and a metal ion-dependent interaction with the CUB module.

##### Free TSG-6 and HC·TSG-6 Utilize Different HA-binding Sites

**FIGURE 7.**
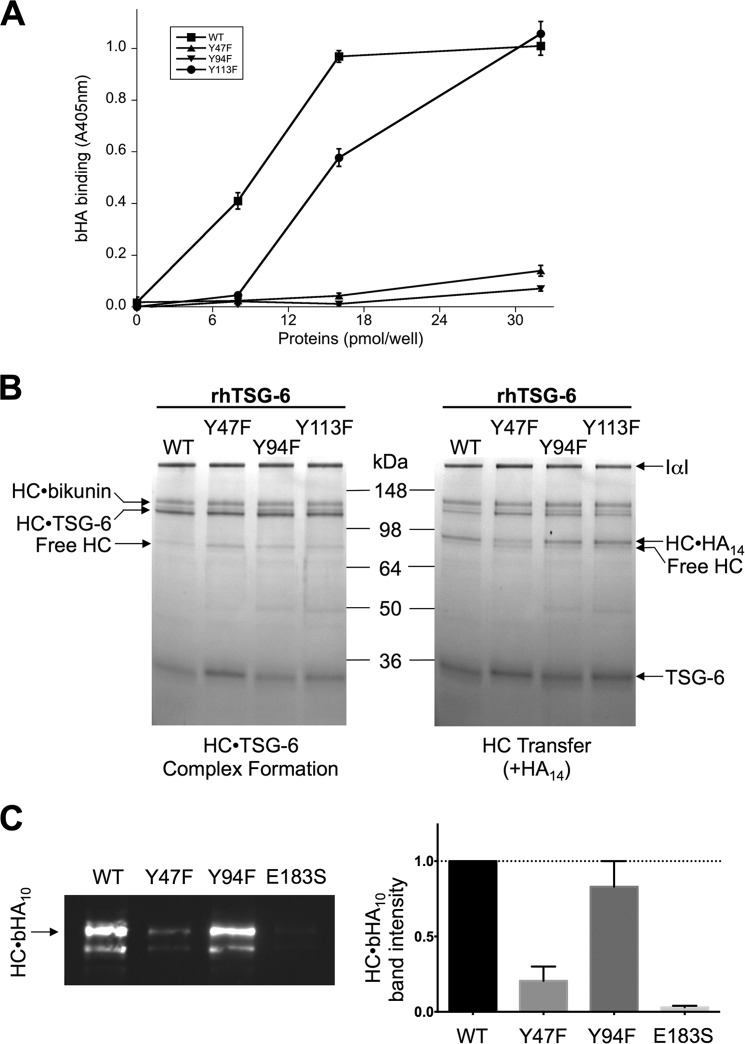
**Determining the role of TSG-6 amino acids in HC·TSG-6 complex formation and HC transfer.** *A*, the HA-binding properties of rhTSG-6 WT and three mutants (Y47F, Y94F, and Y113F) were compared in a microtiter plate-based assay utilizing biotinylated HA (*bHA*); data are mean values ± S.E. (*n* = 8). *B*, SDS-PAGE visualization of HC·TSG-6 complex formation (*left*) and HC transfer (*right*) assays for WT rhTSG-6 and three mutants (with impaired HA-binding activities); in the latter, an HA_14_ oligosaccharide was used as the substrate, leading to the formation of HC·HA_14_ complexes. The gels shown are each representative of three independent experiments. *C*, a bHA_10_ oligosaccharide was used in transfer assays to compare the activities of WT and mutant (Y47F, Y94Y, and E183S) rhTSG-6 proteins, allowing detection of HC·bHA_10_ complexes on blots (*left*) made from gels equivalent to those shown in *B*; bands corresponding to HC·bHA_10_ were quantitated by densitometry (*right*) from three independent experiments (mean ± S.E. (*error bars*)).

As noted in the introduction the formation of HC·HA results from the covalent transfer of a HC from the HC·TSG-6 intermediate onto HA (37). Therefore, it is perhaps not unreasonable to suppose that the HC·TSG-6 complex utilizes the well characterized HA-binding site present in the Link module of TSG-6 (see Ref. 45) for its HA recognition during the transfer process. In order to test this hypothesis, we analyzed three rhTSG-6 mutants with impaired HA-binding activity (Fig. 7*A*) for their ability to form HC·TSG-6 complexes and mediate HC transfer (Fig. 7, *B* and *C*); the three mutants have essentially identical intrinsic fluorescence spectra to the WT rhTSG-6 protein in the absence/presence of reducing and/or denaturing buffer (data not shown), which is consistent with NMR spectroscopy on equivalent mutants in the isolated Link module showing that they all had WT folds (83).

As can be seen from Fig. 7, the Y47F, Y94F, and Y113F mutants have reduced HA-binding activities (Fig. 7*A*), but all retain the ability to form HC·TSG-6 complexes (Fig. 7*B*). However, whereas Y47F has impaired (but not completely abolished) activity for HC transfer (Fig. 7, *B* and *C*), the Y94F and Y113F mutants are indistinguishable from WT rhTSG-6 in their formation of HC·HA (Fig. 7*B*). This provides clear evidence that the HA interaction site in the context of the HC·TSG-6 intermediate is not the same as the HA-binding site in free TSG-6.

**FIGURE 8.**
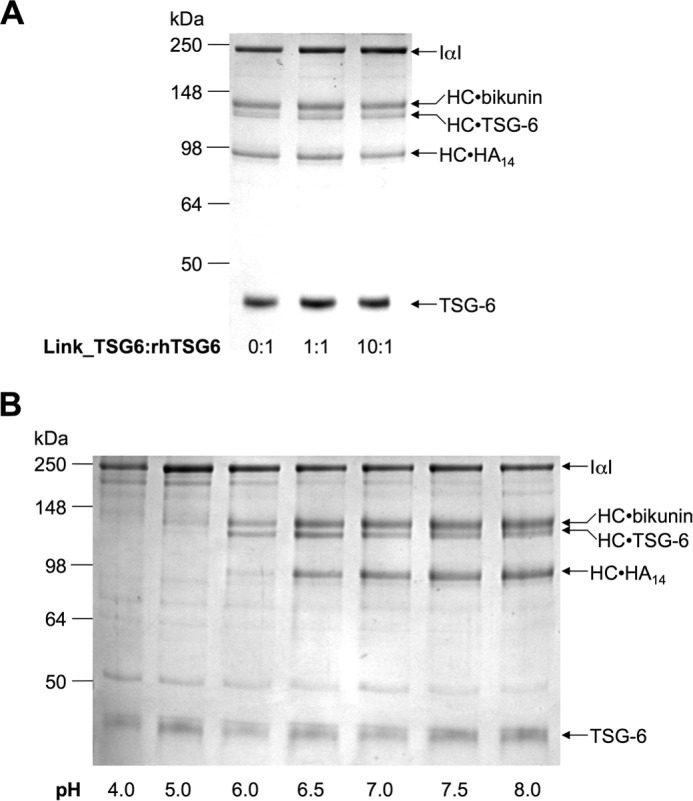
**Role of TSG-6 Link module in HA recognition and pH dependence of complex formation and HC transfer.** *A*, SDS-PAGE analysis of HC transfer assays (with HA_14_ as substrate) in the presence of different molar ratios of Link_TSG6 to rhTSG-6 (at its standard concentration); the gel shown is representative of three independent experiments. *B*, SDS-PAGE analysis of HC transfer assays conducted under different pH conditions with HA_14_ as substrate. These data are representative of three independent experiments.

The above data also reveal that the Link module of TSG-6 contributes directly to HC transfer because the Y47F mutant has impaired activity (Fig. 7, *B* and *C*). However, the addition of excess Link_TSG6 protein did not affect the formation of HC·TSG-6 or HC·HA complexes (Fig. 8*A*), demonstrating that the isolated Link module is not an effective competitive inhibitor of HC transfer.

##### HC·TSG-6 Complex Formation and HC Transfer Have Different pH Dependences

HC transfer assays conducted at a range of pH values between pH 4.0 and 8.0 revealed that the pH dependences for complex formation and HC transfer onto HA are distinct (Fig. 8*B*). At pH 6.0, whereas HC·TSG-6 complexes were clearly formed, only very low levels of HC·HA were detectable; neither of these reactions occurred at pH 4.0, and there was only a faint band observed for the HC·TSG-6 complex at pH 5.0. However, at pH 6.5 (and above), complex formation and HC transfer both occur readily. These data indicate that the individual transesterification reactions that lead to the formation of the HC·TSG-6 intermediate and the HC·HA complex are distinct and probably can be uncoupled. Consistent with this, we showed previously that the covalent HC·TSG-6 complex is stable in the absence of HA for relatively long time periods (at least 26 h) but that the second transesterification can proceed once the HA substrate is provided (37).

##### The HC Transfer Activity of TSG-6 but Not HA Binding Is Necessary for COC Expansion

**FIGURE 9.**
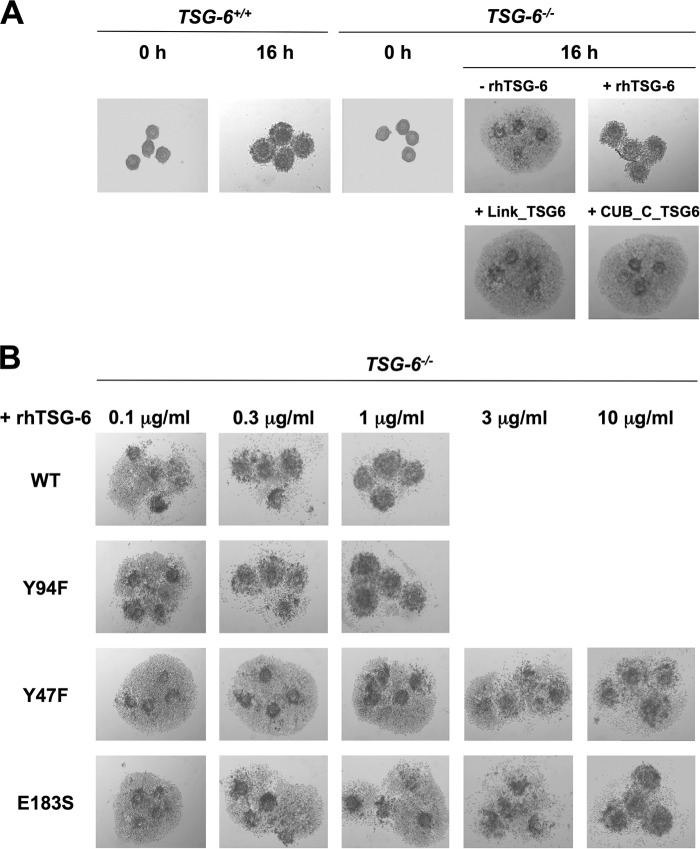
**TSG-6 HC transfer activity rather than its HA binding function plays a critical role in cumulus expansion.** *A*, COCs from *TSG-6*^+/+^ mice undergo cumulus expansion (over 16 h) when stimulated *in vitro* with EGF, but *TSG-6*^−/−^ COCs do not expand, and cumulus cells are shed and settle on the plastic dish. The rhTSG-6 protein (1 μg/ml), but not Link_TSG6 or CUB_C (*CUB_C_TSG6*) (also both at 1 μg/ml), was able to rescue expansion of *TSG-6*^−/−^ COCs. These data are representative of two independent experiments. *B*, expansion of *TSG-6*^−/−^ COCs was completely rescued by the addition of 1 μg/ml WT or Y94F rhTSG-6. Y47F and E183S only had effects at the highest concentration tested (10 μg/ml), where they mediated partial or complete rescue, respectively. The data shown are representative of three independent experiments.

In agreement with previous observations (12), compact COCs isolated from *TSG-6*^−/−^ mice were unable to form an expanded and correctly organized cumulus matrix (Fig. 9*A*) (*i.e.* when stimulated with epidermal growth factor *in vitro* and with serum as the source of IαI). However, the inclusion of 1 μg/ml WT rhTSG-6 protein completely rescued this phenotype such that *TSG-6*^−/−^ COCs expanded in an analogous manner to those from *TSG-6*^+/+^ animals (Fig. 9, *A* and *B*); the isolated Link module and CUB_C domains were unable to rescue cumulus expansion (Fig. 9*A*).

Mutants of rhTSG-6, with differential activities (Fig. 7), were analyzed in this assay system to determine which functions of TSG-6 are required to support the formation of the cumulus extracellular matrix (Fig. 9*B*). Like WT rhTSG-6, the Y94F mutant fully rescued COC expansion in a dose-dependent manner, reaching a maximum effect when added at 1 μg/ml (Fig. 9*B*). This demonstrates that the HA-binding function of TSG-6 is not a major requirement for cumulus expansion because this mutant has greatly impaired HA-binding activity (Fig. 7*A*) while retaining both WT HC·TSG-6 complex formation and HC transfer activities (Fig. 7, *B* and *C*). The E183S mutant, which has impaired (but not abolished) ability to form HC·TSG-6 (Fig. 1*C*) and HC·HA (Fig. 7*C*) complexes, was much less active than WT rhTSG-6, only rescuing the *TSG-6*^−/−^ phenotype when added at 10 μg/ml. The Y47F mutant had lower activity still, only partially rescuing expansion at the highest concentration tested (Fig. 9*B*); this mutant has defective HA binding and impaired HC transfer activity while retaining the ability to form the HC·TSG-6 complex (Fig. 7). Overall, the above data indicate that TSG-6-mediated formation of HC·HA complexes (via HC·TSG-6 intermediates) is critical for the formation of the cumulus matrix, whereas the HA-binding activity of TSG-6 in the context of the free protein does not contribute greatly to this process.

## Discussion

Here, through combined structural and biophysical approaches, we have determined the role of metal ions in the formation of covalent HC·TSG-6 complexes that act as intermediates in HC transfer onto HA. We have also found that although the TSG-6-mediated formation of HC·HA complexes is essential for the expansion of mouse COCs *in vitro*, the HA-binding function of TSG-6 does not play a major role in the stabilization of the murine cumulus matrix.

**FIGURE 10.**
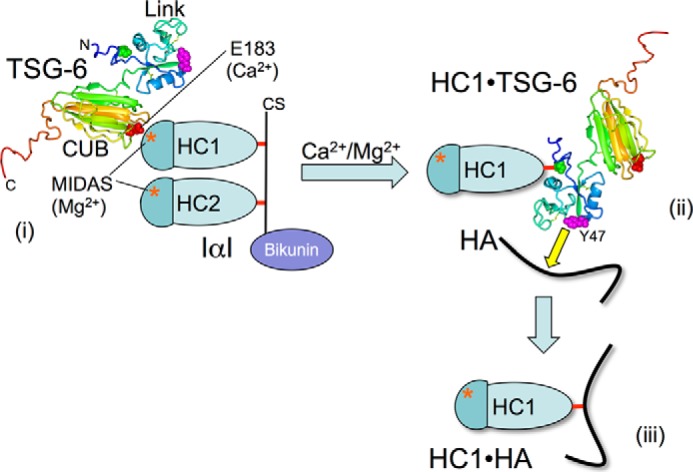
**Schematic model of the metal ion-dependent interaction of TSG-6 with IαI, leading to the formation of HC·HA complexes via an HC·TSG-6 intermediate.** *i*, the CUB_C domain of TSG-6 interacts via its Glu-183 residue (*red space filling*) with the MIDAS site (*) of the HCs (illustrated for HC1) in a metal ion-dependent manner, leading to the formation of an initial non-covalent HC·TSG-6 complex. The Glu-183 amino acid conformation (and the surrounding structure) is stabilized by the presence of bound Ca^2+^, which is essential for the interaction with HCs. *ii*, the non-covalent HC·TSG-6 complex then converts to a covalent complex via the formation of an ester bond (*red*) between Ser-28 of TSG-6 (*green space filling*) and the C-terminal aspartic acid residue of an HC (39). HA is recognized (*yellow arrow*) by a composite surface involving Tyr-47 of the TSG-6 Link module (*pink*) and residues of the HC, leading to the covalent transfer of the HC from TSG-6 onto HA. Previous studies have demonstrated that HC transfer is a divalent cation-dependent process (37), and therefore it is likely to involve the metal ion-dependent interaction in *i* to stabilize the enzyme complex. *iii*, formation of HC·HA leads to release of TSG-6 (not shown), which can then interact with a new IαI molecule and catalyze the formation of further HC·HA.

As illustrated in Fig. 10, HC·TSG-6 complex formation is a divalent cation-dependent reaction (37, 40), which our data reveal is mediated by Ca^2+^, bound to a site within the TSG-6 CUB module. The Glu-183 residue, which is involved in chelating the Ca^2+^ ion, makes a major contribution to the non-covalent interaction with HCs of IαI (*e.g.* via their Mg^2+^-containing MIDAS motifs), which precedes formation of the covalent bond between TSG-6 and HC. Tyr-47 in the Link module of TSG-6 then contributes to HA recognition by the HC·TSG-6 complex during “HC transfer,” but other residues implicated previously in HA binding in free TSG-6 (Tyr-94 and Tyr-113) are not involved in this process.

From the results presented here, it is apparent that Ca^2+^ ion binding is not necessary for the CUB module to fold, but rather it plays a role in providing local structural organization (Figs. 1 and 3) (*e.g.* of surrounding loops). In particular, it probably orients the functionally important Glu-183 amino acid residue, allowing it to interact with the HC. In this regard, our recent crystal studies on human HC1 have revealed that its von Willebrand factor A domain contains a Mg^2+^-containing MIDAS motif, where mutation of the chelating Asp-298 residue abolishes metal ion binding without having any effect on the overall structure.^4^ As shown here, the D298A mutant of rHC1 also abolishes binding to the TSG-6 CUB_C domain (Table 2), providing compelling evidence that this Mg^2+^ ion has a critical function in HC·TSG-6 formation. These findings suggest the intriguing possibility that the metal ion-dependent interaction of TSG-6 with IαI may be mediated by the Glu-183 side chain carboxylate (of the CUB module) co-chelating the magnesium ion within the MIDAS of the HC (*i.e.* reminiscent of interactions between von Willebrand factor A domains of integrins and their RGD-containing ligands (90)).

The discovery that TSG-6 has a Ca^2+^ ion-binding site explains why previously we found it unnecessary to add any calcium to form HC·TSG-6 and HC·HA complexes *in vitro* (37, 45). This is because the rhTSG-6 used in these assays already contained calcium (*i.e.* based on “as purified” CUB_C being fully calcium ion-bound) (Fig. 3). Other studies did, however, indicate a requirement of Ca^2+^ for complex formation and HC transfer (41, 42). In our assay system, the addition of Mg^2+^ ions is required when we are using preparations of IαI where metal ions have been removed during purification (83). Importantly, differences in the sources of protein reagents and assay conditions probably explain the lack of consistency in the conclusions reached previously on the role of metal ions in the formation of HC·TSG-6 and HC·HA (37, 40–42).

Our interaction analyses described here indicate that the full-length TSG-6 can interact with HC1 at two distinct sites: a metal ion-independent interaction with the Link domain and a metal ion-dependent interaction with the CUB module (Fig. 5). Interestingly, both interactions have *K_d_* values of ∼2 nm (Table 2), which is very similar to the affinity (∼5 nm) for the interaction of rhTSG-6 with rHC1 and rHC2 (44). Therefore, it seems unlikely that simultaneous binding of the CUB and Link modules to HCs can occur (because the affinity for rhTSG-6 would then be considerably higher). In this regard, it is reasonable to suggest that of these it is the CUB-mediated (Ca^2+^ ion- and Glu-183-dependent) interaction that is critical for HC·TSG-6 complex formation, given the evidence supporting the role of divalent cations (37, 40–41) and Glu-183 (Figs. 1 and 4*A*) in this process. On the other hand, the interaction with HC via the TSG-6 Link module might provide a mechanism whereby HC·TSG-6 complexes can remain bound to HC·HA and play a role in further catalysis of HC transfer, as suggested previously (44).

TSG-6 can also bind weakly (180 nm) and metal ion-independently to the bikunin·CS component of IαI (40, 46); this is probably mediated (at least in part) through the recognition of the CS chain by the Link module (47). Interestingly, this CS moiety has been clearly implicated as being necessary for HC·TSG-6 formation (40), requiring a particular sulfation pattern in the glycosaminoglycan linkage region in order for IαI to act as a substrate (24); chondroitin (91, 92) and the CS chain of bikunin·CS (93), which has non-sulfated “chondroitin-like” regions (23), can act as weak substrates for HC transfer. At the moment, we do not know the temporal sequence of this CS·bikunin-binding event relative to the metal ion-dependent interaction between the TSG-6 CUB module and an IαI HC (described in the present study). What seems certain is that these interactions are precisely coordinated in such a way as to correctly orient the TSG-6 molecule relative to IαI so that the ester bond connecting an HC to the CS chain can be transferred onto Ser-28 of TSG-6 (39). The fact that HC1·TSG-6 and HC2·TSG-6 complexes form in essentially equal amounts (37, 38) suggests a stochastic element to the process. A plausible mechanism would be for an initial Link module-mediated interaction between the TSG-6 and bikunin·CS, followed by a random “molecular collision” between the Glu-183 of the TSG-6 CUB module and the MIDAS of either HC1 or HC2. This would probably lead to a short lived, high affinity, intermediate involving both interactions, which is destabilized once the HC·CS bond has been transferred onto TSG-6 and the bikunin·CS by-product (37, 38) is released.

Regardless of the precise sequence of the interactions, the non-covalent HC·TSG-6 complex formed must position the Ser-28 side chain of TSG-6 and the HC·CS ester bond in close proximity to the catalytic site, allowing the covalent HC·TSG-6 complex to form via a transesterification reaction (39). However, currently, we do not know where the enzyme active site is located. Our analyses of the pH dependences of complex formation and HC transfer suggest that a histidine (which usually has p*K_a_* values between ∼6.0 and 6.5) is involved in both reactions (Fig. 8*B*); this functional residue(s) is likely to be present in TSG-6, given its role as the catalyst of HC·HA formation (37). In this regard, we observed that residues Asp-200 and His-203 of TSG-6 adopt relative conformations reminiscent of Asp-His-Ser catalytic triads within the CUB module structure, and the propKa software (94, 95) predicted that the *pK_a_* of His-203 might be elevated, as is the case for serine proteases (96). However, the H203S mutant of rhTSG-6 was found to have WT activity for HC·TSG-6 complex formation and HC transfer (data not shown), ruling out a role for this amino acid. Systematic mutagenesis will be required to determine whether a histidine residue of TSG-6 does form part of the catalytic site.

Somewhat counterintuitively, we have found that the HA-binding site in “free” TSG-6 is not the same as that used for HA recognition in the context of HC transfer; this was based on a lack of correlation between the abilities of rhTSG-6 mutants to interact with HA and to form HC·HA complexes (Fig. 7). Furthermore, the observation that the Link_TSG6 protein does not inhibit HC·HA formation (Fig. 8*A*) provided further evidence that the HA-binding site in free TSG-6 is not utilized for HA recognition by HC·TSG-6. This conclusion is consistent with our recent studies showing that there was also no correlation between the substrate activities of various HA oligosaccharides in transfer assays and their affinities for Link_TSG6 (45). Moreover, our previous biophysical experiments have revealed that the interaction of TSG-6 with IαI and the formation of HC·TSG-6 inhibit the binding of TSG-6 to HA, reversing TSG-6-mediated cross-linking of HA (44).

It is noteworthy that the Tyr-47 and Tyr-94 residues are located close together within the HA-binding groove of the TSG-6 Link module (45, 49), yet despite their proximity (∼7 Å between hydroxyl oxygens, based on x-ray structure (50)) and the similar (greatly reduced) HA-binding phenotype of their phenylalanine mutants (Fig. 7*A*), they exhibit markedly different contributions to HC transfer activity (Fig. 7, *B* and *C*). This could be explained by the formation of a composite HA recognition site involving both TSG-6 and HC within the HC·TSG-6 complex, in which Tyr-47, but not Tyr-94 or Tyr-113, of TSG-6 plays a role (*i.e.* where HC probably occludes part of the binding surface used for HA in free TSG-6).

Given that HC·TSG-6 (unlike free TSG-6) does not bind tightly to HA (44), it seems plausible that during HC transfer, there is only a transient “interaction” of HA with the active site of this enzyme complex. It seems likely that residues from both TSG-6 and HC contribute to a composite active site (including a histidine, as discussed earlier) that is stabilized by the Glu-183- and Ca^2+^-dependent interaction of the CUB module with the MIDAS site of the HC (*i.e.* based on the requirement for divalent cations in the transfer of HC onto HA (37)). This active site is probably similar to that used in the initial transesterification reaction (*i.e.* within the non-covalent HC·TSG-6 complex). However, it is clearly not identical, as can be inferred from the different pH minima of the two reactions (Fig. 8*B*); this is to be expected because the formation of HC·HA has a different specificity requirement, with the transfer of the ester bond onto the C6 hydroxyl of HA (28) rather than onto the side chain hydroxyl of Ser-28 (39).

Here we have demonstrated that the TSG-6-dependent transfer of HC onto HA is an absolute requirement for the organization/stabilization of the cumulus matrix, whereas the HA-binding properties of TSG-6 do not play an important role (*i.e.* based on the ability of rhTSG-6 mutants to rescue *in vitro* expansion of COCs from *TSG-6*^−/−^ mice) (Fig. 9). These data are consistent with previous studies showing the involvement of HC·HA in COC expansion (9, 12, 13, 91), where the formation of these complexes is mediated by TSG-6 (37, 39, 40). Furthermore, the present study indicates that TSG-6 is not a major participant in the structural stabilization of the cumulus matrix through its direct cross-linking of HA chains, as has been suggested previously (12, 13, 15, 31, 34, 53, 54). This is perhaps not surprising, given the recent findings that the interaction of TSG-6 with IαI impairs the binding of TSG-6 to HA (44) and that the full-length TSG-6 protein is unable to bridge between pentraxin-3 and HA (16), although this is a property of its isolated Link module domain (15, 16). Moreover, our recent biophysical studies have provided strong evidence that TSG-6, IαI, and pentraxin-3 cooperate to cross-link HA (16), where multiple HC·HA complexes probably associate with the octamer pentraxin-3 (31–33). However, this cross-linking process is tightly regulated, and, surprisingly, pentraxin-3 does not integrate into preformed HC·HA films but requires a prior encounter with IαI (16). Why this should be the case is unclear, indicating that there is still much we do not understand regarding how the process of COC expansion is controlled (both temporally and spatially) and the way that these proteins organize HA in the cumulus matrix. In this regard, it seems probable that as well as the association of HC·HA with pentraxin-3, there are likely to be other interactions that play a role in stabilizing the HA network. Interestingly, the finding that the Y47F mutant of rhTSG-6 has the most impaired rescue activity (Fig. 9*B*), although it retains more HC transfer activity than E183S (Fig. 7*C*), is indicative that TSG-6 does play an additional role in COC expansion besides its catalysis of HC·HA formation.

This research has provided important new insights into the mechanisms underlying TSG-6-mediated HC·HA formation and has clarified the role of divalent metal ions in this fundamental biological process. It has also identified that it is the transferase activity of TSG-6 that is essential for COC expansion rather than its HA-binding function. These studies therefore provide an excellent basis for additional work to further understand the molecular basis of HA cross-linking during ovulation and inflammation.

## Author Contributions

D. C. B. was responsible for protein crystallization, crystallographic data collection, processing and refinement, heavy chain transfer assays, and drafting the paper. H. L. B. performed surface plasmon resonance, intrinsic fluorescence spectroscopy, and CUB_C expression and purification. T. A. carried out CUB_C expression/purification and NMR. M. S. R. conducted mutagenesis of rhTSG-6, heavy chain transfer, and HA-binding assays. J. P. W. aided in NMR data collection, processing, and interpretation. E. I. conducted the *in vitro* COC expansion assays. T. A. J. co-supervised H. L. B. and aided in fitting/interpretation of SPR data. J. J. E. provided purified IαI protein and contributed to writing of the paper. R. P. R. provided the bHA_10_ oligosaccharide and contributed to writing of the paper. A. S. supervised the *in vitro* COC expansion assays and contributed to writing of the paper. C. M. M. co-directed research and contributed to writing of the paper. A. J. D. directed research, supervised experiments, and coordinated writing of the paper.

## Footnotes

4 D. C. Briggs, C. M. Milner, and A. J. Day, unpublished data.

## Footnotes

3 The abbreviations used are:

COC: cumulus-oocyte complex
CS: chondroitin sulfate
CUB: complement C1r/C1s, Uegf, and BMP1
CUB_C: CUB module and C-terminal peptide from human TSG-6
HA: hyaluronan
bHA_10_: HA decasaccharide biotinylated at the reducing end
HC: heavy chain
rHC1 and rHC2: recombinant HC1 and HC2, respectively
rhTSG-6: recombinant human TSG-6
HC·HA: hyaluronan with heavy chains covalently attached
HC·TSG-6: covalent complex of TSG-6 and a heavy chain
IαI: inter-α-inhibitor
Link_TSG6: recombinant Link module from human TSG-6
SPR: surface plasmon resonance
Tricine: *N*-[2-hydroxy-1,1-bis(hydroxymethyl)ethyl]glycine
MIDAS: metal ion-dependent adhesion site.
